# Pressure Ulcer Prevention in the Hospital Setting Using Silicone Foam Dressings

**DOI:** 10.7759/cureus.730

**Published:** 2016-08-08

**Authors:** Bao Truong, Eileen Grigson, Maulik Patel, Xinwei Liu

**Affiliations:** 1 University of Central Florida College of Medicine

**Keywords:** pressure ulcers, silicone dressings, preventive medicine, hospital acquired infection, pressure sores, decubitus ulcer

## Abstract

Patient care is of the utmost importance in the hospital setting. Bedrest and immobility during hospitalization, especially in the surgical and intensive care setting, place the patient at high risk for pressure ulcers. It is very important to prevent or notice a pressure ulcer forming due to the significant health care costs involved and patient health associated with them. Various measures are in place to prevent patients from getting pressure ulcers, but a newer material, silicone foam dressings, has been introduced as an alternative solution for the prevention of these ulcers. We review the current literature to examine whether the standard protocol or silicone material is superior to the prevention of pressure ulcer formation. We conclude that silicone foam dressings, when used as prophylactic treatment, seems very promising and may even be superior to the standard care of prevention. However, there were limitations to some studies and further research is needed to confirm the role of silicone foam dressings.

## Introduction and background

Pressure ulcers, also called pressure sores or bedsores, are a burden to healthcare and have a significant cost of morbidity. In 2013, a United States Medicare study reported the incidence of hospital-acquired pressure ulcers to be 4.5% in hospitalized patients, with an estimated 11 billion dollars for the cost of pressure ulcer care [1-2]. 

Pressure ulcers are due to a multitude of factors that contribute to tissue vulnerability and breakdown. In 2007, the National Pressure Ulcer Advisory Panel (NPUAP) established a staging system for categorizing pressure ulcer injuries. Pressure ulcers are often formed where skin covers bony areas and common sites are the back of the head and ears, the shoulders, the elbows, the lower back and buttocks, the hips, the inner knees, and the heels. Although pressure ulcers can develop over the course of 24 hours, they may not present until a week later. Stage I pressure ulcers present with intact, erythematous skin that does not blanch. Stage II pressure ulcers can appear as a fluid-filled blister, which represents breakage of the epidermis and may involve the underlying dermis. Stage III pressure ulcers present with necrotic tissue and extend into the subcutaneous tissue. Finally, Stage IV pressure ulcers extend deep into the bone or muscle with full thickness tissue loss [3].

The incidence and prevalence of pressure ulcers vary greatly, depending on the setting. Patients hospitalized in the intensive care unit (ICU) or immobilized after major surgery are at higher risk [4]. Vigilant patient care teams educated in pressure ulcer care can identify the signs of tissue breakdown and are aware of the numerous factors that place patients at risk of pressure ulcer formation, including reduced mobility, nutritional status, urine or fecal incontinence, medications that cause reduced sensation and immobility, instruments and tools that create mechanical pressure against the body, and conditions that decrease tissue oxygenation [3].

Barrier creams used in the prevention of pressure ulcers form a protective layer that keeps away excessive moisture due to incontinence, perspiration, or wound drainage and aid in maintaining the integrity of the skin [5]. These creams include Calmoseptine® (Calmoseptine, Inc., Huntington Beach, CA), Lantiseptic® (Santus, Duluth, GA), Silvadene® (Pfizer, Inc., New York, NY), and others that use silver sulfadiazine, zinc oxide, or lanolin as active ingredients to prevent infection. A hydrocolloid dressing normally used in wound care, such as Tegaderm® (3M Center, St. Paul, MN), is also frequently used in combination with barrier creams and contains an adhesive compound in combination with a water-resistant outer layer to prevent additional moisture exposure [6]. Silicone foam dressings, such as Mepilex® (Mölnlycke Health Care, Gothenburg, Sweden), are soft silicone multi-layered foam dressings that contain silver, which acts as an antibiotic agent, and an adhesive material different from the regularly used hydrocolloids that result in less skin abrasion when it is time to remove and replace the dressing [7].

Currently, standard protocols for care in pressure ulcer prevention vary between hospital systems with different algorithms for inpatient and outpatient situations but include the use of low-pressure beds, positioning and turning, and barrier cream with a hydrocolloid layering placed over the area of application in areas at risk of pressure formation, such as the sacrum, heels, and buttocks [3]. Although the standard care has been effective, a newer material, silicone foam dressing, has been introduced as an alternative for pressure ulcer prevention with potentially greater cost and health benefits for hospitals and patients, respectively [8]. If the silicone foam dressings are, indeed, better at treating and preventing sore formation in immobilized patients, then nurses should also benefit in their role as patient caregivers by being able to provide greater and more efficient care to their patients.

This literature review will examine whether immobilized patients in the hospital setting who are given silicone foam dressings compared to the standard protocol, which utilizes barrier creams under a hydrocolloid layering for the prevention of pressure ulcer, have an effect on the incidence of Stage I pressure ulcer formation.

## Review

A literature review was conducted to determine the effectiveness of standard protocols for pressure ulcer care versus a newer silicone foam dressing. PubMed searches were performed using the phrases “silicone foam dressing” and “barrier creams” in the English language with the modifier of articles published in the last seven years. Articles were then screened for relevance and excluded if the studies were not primarily focused on pressure ulcer prevention. This search process yielded five quantitative research articles focusing on the usage and effectiveness of silicone foam dressings.

This review covers the five separate studies at various institutions detailing the utility and benefits of using silicone foam dressing as an alternative to the standard care of pressure ulcer prevention (Table 1). Huang, et al. sought to determine if there was any way to reduce the incidence of nasal pressure ulcers that arise as a complication of nasotracheal intubation during oral and maxillofacial surgery [6]. By using an initial animal model to test the clinical application of silicone foam material as a means of reducing pressure on the nasal area, Huang, et al. believed that the use of the cushioning material would aid in protection during intubation as opposed to intubation without additional cushion protection. Eighteen patients were studied. The results showed a decrease in the size of nasal pressure sores between the control and comparison groups to which Huang, et al. attributed to the protective efficacy of the silicone foam dressing used as lining during nasal intubation. It should be noted, however, that only a small sample size of 18 participants was included in this study.

**Table 1:** Study Characterisitics n = number

Publication Date, Authors	Location, Setting, *n*	Body Site Reported/Stage of Ulcer	Study Design	Pressure Ulcer Prophylactic	Findings
(2009) Huang, et al. [6]	Dalin, Taiwan, Republic of China, Buddhist Dalin Tzu Chi General Hospital operating room, n = 18	Nose/ Stage I	Quantitative prospective cohort study. Patients in the study group had foam surrounding their intubation tubes compared to the control group who did not.	Silicone foam dressing vs standard hospital treatment protocol from Tzu Chi General Hospital.	Silicone foam dressings were found to reduce the incidence of pressure ulcer formation due to nasotracheal tube intubation. 8/8 (100%) formed pressure ulcers in the control group while 6/10 (60%) in the intervention group formed pressure ulcers.
(2011) Forni, et al. [9]	Italy, Rizzoli Orthopedic Institute, n = 105	Heel/ Stage I	Quantitative prospective cohort study. Patients in the intervention group were compared to control group data collected in the previous year.	Silicone foam dressing vs standard hospital treatment protocol from Rizzoli Orthopedic Institute.	Pressure ulcer reduction in patients wearing casts was possible using silicone foam dressings placed within the cast with 2/56 (3.6%) forming pressure ulcers compared to the control group incidence rate of 21/49 (42.9%).
(2012) Brindle and Wegelin [4]	Virginia, Virginia Commonwealth University Medical Center ICU, n = 85	Sacrum/Stage I	Quantitative prospective cohort study. Intervention group was given Mepilex® Border Sacrum dressings with standard care protocol compared to control group, which only received standard care protocol	Silicone foam dressing vs standard hospital treatment protocol from Virginia Commonwealth University Medical Center	Patients undergoing cardiac surgery who were given silicone foam dressings after surgery had pressure ulcer formation incidence of 1/50 (2%) compared to the control group who did not receive the silicone foam dressing 4/35 (11.4%). The findings were not statistically significant, however, due to sample size.
(2012) Chaiken [11]	Illinois, Swedish Covenant Hospital ICU, n = 563	Sacrum/Stage I	Quantitative prospective cohort study. Patients in the study group were compared to those in the control group data collected the previous year.	Silicone foam dressing vs standard hospital treatment protocol from Swedish Covenant Hospital.	Reduction of sacral pressure ulcers was found in the intervention group with 5/273 (1.8%) incidence of pressure ulcer formation compared to 36/291 (12.3%) in the control group.
(2015) Santamaria, et al. [12]	Australia, Royal Melbourne Hospital ICU n = 313	Sacrum/ heel/Stage I	Randomized controlled trial with the intervention group receiving Mepilex® Border Sacrum and Mepilex® Heel dressings. Both groups received standard prevention strategies.	Silicone foam dressing vs standard hospital treatment protocol from Royal Melbourne Hospital.	There was a significantly decreased formation of pressure ulcers in the intervention group in comparison to the control group who received traditional wound dressing. 5/161 (3.1%) developed pressure ulcers in the intervention group vs 20/152 (13.1%) in the control group.

Forni, et al. investigated pressure ulcer prevention by using foam dressings in patients with cast immobilization [9]. One hundred and fifty-six patients were included in this study, 85 in the control group and 71 in the experimental group. Inclusion criteria included any patient admitted to an Italian orthopedic research hospital for an orthopedic associated disease requiring a plaster cast of their foot. The results provided substantial evidence of pressure ulcer prevention by applying foam within the plaster cast of patients with data that was statistically significant for differences among the control and experimental groups. Forni, et al. discussed the positive impact that foams can have as a deterrent against pressure ulcers in patients requiring casts as well as its potential use in diabetics who are more prone to developing pressure ulcers [9].

Cardiac surgery patients are one of the most at-risk patient populations in the incidence of hospital-acquired pressure ulcers [10]. In 2012, Brindle and Wegelin hypothesized that using Mepilex®, a silicone foam dressing, around the area of the sacrum in these patients would decrease incidence rates of pressure ulcer formation in comparison to the hospital’s current standard care, which consisted of the use of a skin protectant, Calmoseptine®, on high risk areas [4]. Silicone foam dressing impregnated with silver as an antibiotic was the independent variable in the study, while the dependent variable was the formation of pressure ulcers in the area of the sacrum. A total of 85 patients were enrolled in this study who had met the criterion for inclusion and exclusion. Eligibility for the study included patients who had undergone a surgical procedure within a certain period of time, cardiac arrest on admission, in shock, or had some sort of condition that required chronic bed rest. Statistical analyses for the variables collected showed no significant differences when compared between the experimental versus the control groups. Results showed that the incidence rate of pressure ulcer formation in patients who were given Mepilex® silicone foam dressing was not considerably less than in patients who were given the barrier cream. Brindle and Wegelin concluded in their paper that, while using silicone foam dressings did produce a decreased incidence rate in pressure ulcers in the intervention group, it was not by much in comparison to the control group. They reasoned that the smaller sample size than what was originally planned, in addition to the standard care procedures and attentive efforts of the ICU nurses in patient treatment, may have played a part in the findings of the study.

In 2012, Chaiken, in a similar study, sought to determine whether the incidence of pressure ulcer formation would decrease in the ICU through the use of silicone foam dressing placed at the area of the sacrum [11]. During the prospective experiment, Mepilex® Border Sacrum, a type of silicone foam, was given to a group of 273 newly admitted patients in the ICU with nurses instructed in changing the dressing twice a week while following the hospital's standard protocol for pressure ulcer prevention. The study took place over a six-month period and the results showed a decrease in the incidence rates of pressure ulcers from 12.3% during the 35-month observation period in the comparison group to 1.8% in the experiment group. In addition to the lower incidence rate of pressure ulcers, Chaiken further reported an average expenditure of $6,653.00 for the six-month experiment period, which, in turn, saved the hospital money by avoiding the higher cost of treatment associated with treating pressure ulcers.

In 2013, Santamaria, et al. examined the effectiveness of soft silicone dressings of the sacrum and the heel of the body in the ICU setting in preventing pressure ulcers [12]. This randomized control study included 440 patients who were newly admitted to the emergency department and were subsequently transferred to the ICU. Findings were significant for decreased formation of pressure ulcers in the intervention group, as measured by the Australian Wound Management Association (AWMA) four-point grading system, in comparison to the control group who received a standard barrier cream with a hydrocolloid layering. Santamaria, et al*.* concluded that use of silicone foam dressings in combination with thorough risk assessment and evidence-based pressure ulcer prevention strategies had a substantial impact on the reduction of pressure ulcer incidence.

Upon review of the articles, there is efficacy and utility of silicone foam dressings among other forms of barriers in the prevention of pressure ulcers. This literature review shows that standard traditional methods were helpful in preventing pressure ulcers, but silicone foam dressings were even more effective in reducing the incidence of pressure ulcer formation (3.6% versus 42.9% in the study conducted by Forni, et al.) [9]. Not only should silicone foam dressings be considered due to their effectiveness, Santamaria, et al. concluded in their study that they should be considered due to their economic savings to health care institutions (average net cost of intervention compared to control, $52.87 versus $107.91) [12]. There were still limitations, which were evident when reviewing the literature, even though each article provided evidence regarding the effectiveness of silicone foam dressings. Research conducted by Huang, et al. provided information regarding protection of pressure ulcer formation, but the paper did not include the methods of data collection or methods of analyses [6]. The research by Forni, et al. and Chaiken was limited by a lack of a parallel control within these respective studies and used past patient data as a control instead [9, 11]. Brindle and Wegelin mentioned that a major limitation of their study was indeed the duration of the experiment and sample size [4].

## Conclusions

We would like to recommend that future research would not only benefit from increasing the sample size and diversifying the population but should strive to conduct trials over longer periods of time in order to obtain adequate data and use the maximal amount of data it can for analysis. Additionally, the research may be expanded to other clinical areas where the incidence of pressure ulcer formation is high, such as patients with incontinence, recurrent pressure ulcers, and the elderly hospitalized with altered mental status.

Proper skin care is very important in the prevention of these ulcers. Even though guidelines are in place for the prevention of pressure ulcers, the incidence is still very high in patients who are in the ICU or who have had major surgeries. Prevention is extremely important because not only does the patient suffer from the pressure ulcer, but there is an economic impact related to them as the hospital may incur additional costs related to pressure ulcer management. In addition to standard protocols, the use of silicone foam dressings as a barrier against irritation and constant pressure to the skin should be effectively utilized in pressure ulcer prevention.
